# Supplementary material for: HIVprotI: an integrated web based platform for prediction and design of HIV proteins inhibitors
Source: J Cheminform. 2018 Mar 9;10:12. doi: 10.1186/s13321-018-0266-y (PMC5845081; doi:10.1186/s13321-018-0266-y)
Supplement: Supplementary file 2 — Additional file 2. Source code of HIVProtI web server. [file 13321_2018_266_MOESM2_ESM.zip › HIVprotI_Source-code/hivprotI/weka-3-6-6/doc/weka/classifiers/bayes/net/search/global/GlobalScoreSearchAlgorithm.html]

GlobalScoreSearchAlgorithm


---


|  |  |  |
| --- | --- | --- |
| **Overview** \| **Package** \| **Class** \| **Tree** \| **Deprecated** \| **Index** \| **Help** \| **Weka's home** | |  |
| **PREV CLASS**   **NEXT CLASS** | **FRAMES**    **NO FRAMES**     **All Classes** |  |
| SUMMARY: NESTED \| FIELD \| CONSTR \| METHOD | DETAIL: FIELD \| CONSTR \| METHOD |  |


---


## weka.classifiers.bayes.net.search.global Class GlobalScoreSearchAlgorithm

```
java.lang.Object
  weka.classifiers.bayes.net.search.SearchAlgorithm
      weka.classifiers.bayes.net.search.global.GlobalScoreSearchAlgorithm
```

**All Implemented Interfaces:**: java.io.Serializable, OptionHandler, RevisionHandler

**Direct Known Subclasses:**: GeneticSearch, HillClimber, K2, SimulatedAnnealing, TAN

---

``` public class GlobalScoreSearchAlgorithm extends SearchAlgorithm ```

This Bayes Network learning algorithm uses cross validation to estimate classification accuracy.


Valid options are:

```
 -mbc
  Applies a Markov Blanket correction to the network structure, 
  after a network structure is learned. This ensures that all 
  nodes in the network are part of the Markov blanket of the 
  classifier node.
```

```
 -S [LOO-CV|k-Fold-CV|Cumulative-CV]
  Score type (LOO-CV,k-Fold-CV,Cumulative-CV)
```

```
 -Q
  Use probabilistic or 0/1 scoring.
  (default probabilistic scoring)
```

**Version:**
:   $Revision: 1.10 $

**Author:**
:   Remco Bouckaert

**See Also:**: Serialized Form

---

| **Field Summary** | |
| --- | --- |
| `static Tag[]` | `TAGS_CV_TYPE`             the score types |


**Constructor Summary**

`GlobalScoreSearchAlgorithm()`


| **Method Summary** | |
| --- | --- |
| `double` | `calcScore(BayesNet bayesNet)`             performCV returns the accuracy calculated using cross validation. |
| `double` | `calcScoreWithExtraParent(int nNode, int nCandidateParent)`             Calc Node Score With Added Parent |
| `double` | `calcScoreWithMissingParent(int nNode, int nCandidateParent)`             Calc Node Score With Parent Deleted |
| `double` | `calcScoreWithReversedParent(int nNode, int nCandidateParent)`             Calc Node Score With Arrow reversed |
| `double` | `cumulativeCV(BayesNet bayesNet)`             CumulativeCV returns the accuracy calculated using cumulative cross validation. |
| `java.lang.String` | `CVTypeTipText()` |
| `SelectedTag` | `getCVType()`             get cross validation strategy to be used in searching for networks. |
| `boolean` | `getMarkovBlanketClassifier()` |
| `java.lang.String[]` | `getOptions()`             Gets the current settings of the search algorithm. |
| `java.lang.String` | `getRevision()`             Returns the revision string. |
| `boolean` | `getUseProb()` |
| `java.lang.String` | `globalInfo()`             This will return a string describing the search algorithm. |
| `double` | `kFoldCV(BayesNet bayesNet, int nNrOfFolds)`             kFoldCV uses k-fold cross validation to measure the accuracy of a Bayes network classifier. |
| `double` | `leaveOneOutCV(BayesNet bayesNet)`             LeaveOneOutCV returns the accuracy calculated using Leave One Out cross validation. |
| `java.util.Enumeration` | `listOptions()`             Returns an enumeration describing the available options |
| `java.lang.String` | `markovBlanketClassifierTipText()` |
| `void` | `setCVType(SelectedTag newCVType)`             set cross validation strategy to be used in searching for networks. |
| `void` | `setMarkovBlanketClassifier(boolean bMarkovBlanketClassifier)` |
| `void` | `setOptions(java.lang.String[] options)`             Parses a given list of options. |
| `void` | `setUseProb(boolean useProb)` |
| `java.lang.String` | `useProbTipText()` |

**Methods inherited from class weka.classifiers.bayes.net.search.SearchAlgorithm**

`buildStructure, initAsNaiveBayesTipText, maxNrOfParentsTipText, toString`

**Methods inherited from class java.lang.Object**

`equals, getClass, hashCode, notify, notifyAll, wait, wait, wait`

**Field Detail**

### TAGS\_CV\_TYPE

```
public static final Tag[] TAGS_CV_TYPE
```

:   the score types


**Constructor Detail**

### GlobalScoreSearchAlgorithm

```
public GlobalScoreSearchAlgorithm()
```


**Method Detail**

### calcScore

```
public double calcScore(BayesNet bayesNet)
                 throws java.lang.Exception
```

:   performCV returns the accuracy calculated using cross validation.
    The dataset used is m\_Instances associated with the Bayes Network.

    :   **Parameters:**: `bayesNet` - : Bayes Network containing structure to evaluate **Returns:**: accuracy (in interval 0..1) measured using cv. **Throws:**: `java.lang.Exception` - whn m\_nCVType is invalided + exceptions passed on by updateClassifier

---


### calcScoreWithExtraParent

```
public double calcScoreWithExtraParent(int nNode,
                                       int nCandidateParent)
                                throws java.lang.Exception
```

:   Calc Node Score With Added Parent

    :   **Parameters:**: `nNode` - node for which the score is calculate: `nCandidateParent` - candidate parent to add to the existing parent set **Returns:**: log score **Throws:**: `java.lang.Exception` - if something goes wrong

---


### calcScoreWithMissingParent

```
public double calcScoreWithMissingParent(int nNode,
                                         int nCandidateParent)
                                  throws java.lang.Exception
```

:   Calc Node Score With Parent Deleted

    :   **Parameters:**: `nNode` - node for which the score is calculate: `nCandidateParent` - candidate parent to delete from the existing parent set **Returns:**: log score **Throws:**: `java.lang.Exception` - if something goes wrong

---


### calcScoreWithReversedParent

```
public double calcScoreWithReversedParent(int nNode,
                                          int nCandidateParent)
                                   throws java.lang.Exception
```

:   Calc Node Score With Arrow reversed

    :   **Parameters:**: `nNode` - node for which the score is calculate: `nCandidateParent` - candidate parent to delete from the existing parent set **Returns:**: log score **Throws:**: `java.lang.Exception` - if something goes wrong

---


### leaveOneOutCV

```
public double leaveOneOutCV(BayesNet bayesNet)
                     throws java.lang.Exception
```

:   LeaveOneOutCV returns the accuracy calculated using Leave One Out
    cross validation. The dataset used is m\_Instances associated with
    the Bayes Network.

    :   **Parameters:**: `bayesNet` - : Bayes Network containing structure to evaluate **Returns:**: accuracy (in interval 0..1) measured using leave one out cv. **Throws:**: `java.lang.Exception` - passed on by updateClassifier

---


### cumulativeCV

```
public double cumulativeCV(BayesNet bayesNet)
                    throws java.lang.Exception
```

:   CumulativeCV returns the accuracy calculated using cumulative
    cross validation. The idea is to run through the data set and
    try to classify each of the instances based on the previously
    seen data.
    The data set used is m\_Instances associated with the Bayes Network.

    :   **Parameters:**: `bayesNet` - : Bayes Network containing structure to evaluate **Returns:**: accuracy (in interval 0..1) measured using leave one out cv. **Throws:**: `java.lang.Exception` - passed on by updateClassifier

---


### kFoldCV

```
public double kFoldCV(BayesNet bayesNet,
                      int nNrOfFolds)
               throws java.lang.Exception
```

:   kFoldCV uses k-fold cross validation to measure the accuracy of a Bayes
    network classifier.

    :   **Parameters:**: `bayesNet` - : Bayes Network containing structure to evaluate: `nNrOfFolds` - : the number of folds k to perform k-fold cv **Returns:**: accuracy (in interval 0..1) measured using leave one out cv. **Throws:**: `java.lang.Exception` - passed on by updateClassifier

---


### getUseProb

```
public boolean getUseProb()
```

:   **Returns:**: use probabilities or not in accuracy estimate

---


### setUseProb

```
public void setUseProb(boolean useProb)
```

:   **Parameters:**: `useProb` - : use probabilities or not in accuracy estimate

---


### setCVType

```
public void setCVType(SelectedTag newCVType)
```

:   set cross validation strategy to be used in searching for networks.

    :   **Parameters:**: `newCVType` - : cross validation strategy

---


### getCVType

```
public SelectedTag getCVType()
```

:   get cross validation strategy to be used in searching for networks.

    :   **Returns:**: cross validation strategy

---


### setMarkovBlanketClassifier

```
public void setMarkovBlanketClassifier(boolean bMarkovBlanketClassifier)
```

:   **Parameters:**: `bMarkovBlanketClassifier` -

---


### getMarkovBlanketClassifier

```
public boolean getMarkovBlanketClassifier()
```

:   **Returns:**

---


### listOptions

```
public java.util.Enumeration listOptions()
```

:   Returns an enumeration describing the available options

    :   **Specified by:**: `listOptions` in interface `OptionHandler` **Overrides:**: `listOptions` in class `SearchAlgorithm`
    :   **Returns:**: an enumeration of all the available options

---


### setOptions

```
public void setOptions(java.lang.String[] options)
                throws java.lang.Exception
```

:   Parses a given list of options.


    Valid options are:

    ```
     -mbc
      Applies a Markov Blanket correction to the network structure, 
      after a network structure is learned. This ensures that all 
      nodes in the network are part of the Markov blanket of the 
      classifier node.
    ```

    ```
     -S [LOO-CV|k-Fold-CV|Cumulative-CV]
      Score type (LOO-CV,k-Fold-CV,Cumulative-CV)
    ```

    ```
     -Q
      Use probabilistic or 0/1 scoring.
      (default probabilistic scoring)
    ```

    :   **Specified by:**: `setOptions` in interface `OptionHandler` **Overrides:**: `setOptions` in class `SearchAlgorithm`
    :   **Parameters:**: `options` - the list of options as an array of strings **Throws:**: `java.lang.Exception` - if an option is not supported

---


### getOptions

```
public java.lang.String[] getOptions()
```

:   Gets the current settings of the search algorithm.

    :   **Specified by:**: `getOptions` in interface `OptionHandler` **Overrides:**: `getOptions` in class `SearchAlgorithm`
    :   **Returns:**: an array of strings suitable for passing to setOptions

---


### CVTypeTipText

```
public java.lang.String CVTypeTipText()
```

:   **Returns:**: a string to describe the CVType option.

---


### useProbTipText

```
public java.lang.String useProbTipText()
```

:   **Returns:**: a string to describe the UseProb option.

---


### globalInfo

```
public java.lang.String globalInfo()
```

:   This will return a string describing the search algorithm.

    :   **Returns:**: The string.

---


### markovBlanketClassifierTipText

```
public java.lang.String markovBlanketClassifierTipText()
```

:   **Returns:**: a string to describe the MarkovBlanketClassifier option.

---


### getRevision

```
public java.lang.String getRevision()
```

:   Returns the revision string.

    :   **Specified by:**: `getRevision` in interface `RevisionHandler` **Overrides:**: `getRevision` in class `SearchAlgorithm`
    :   **Returns:**: the revision


---


|  |  |  |
| --- | --- | --- |
| **Overview** \| **Package** \| **Class** \| **Tree** \| **Deprecated** \| **Index** \| **Help** \| **Weka's home** | |  |
| **PREV CLASS**   **NEXT CLASS** | **FRAMES**    **NO FRAMES**     **All Classes** |  |
| SUMMARY: NESTED \| FIELD \| CONSTR \| METHOD | DETAIL: FIELD \| CONSTR \| METHOD |  |


---
